# Shared Genes of *PPARG* and *NOS2* in Alzheimer’s Disease and Ulcerative Colitis Drive Macrophages and Microglia Polarization: Evidence from Bioinformatics Analysis and Following Validation

**DOI:** 10.3390/ijms24065651

**Published:** 2023-03-15

**Authors:** Longcong Dong, Yuan Shen, Hongying Li, Ruibin Zhang, Shuguang Yu, Qiaofeng Wu

**Affiliations:** 1 Acupuncture and Tuina College, Chengdu University of Traditional Chinese Medicine, Chengdu 610075, China; 2 Acupuncture & Chronobiology Key Laboratory of Sichuan Province, Chengdu 610075, China

**Keywords:** Alzheimer’s disease, ulcerative colitis, *PPARG*, *NOS2*, shared gene, bioinformatics

## Abstract

Emerging evidence shows that peripheral systemic inflammation, such as inflammatory bowel disease (IBD), has a close even interaction with central nervous disorders such as Alzheimer’s disease (AD). This study is designed to further clarify the relationship between AD and ulcerative colitis (UC, a subclass of IBD). The GEO database was used to download gene expression profiles for AD (GSE5281) and UC (GSE47908). Bioinformatics analysis included GSEA, KEGG pathway, Gene Ontology (GO), WikiPathways, PPI network, and hub gene identification. After screening the shared genes, qRT-PCR, Western blot, and immunofluorescence were used to verify the reliability of the dataset and further confirm the shared genes. GSEA, KEGG, GO, and WikiPathways suggested that *PPARG* and *NOS2* were identified as shared genes and hub genes by cytoHubba in AD and UC and further validated via qRT-PCR and Western blot. Our work identified *PPARG* and *NOS2* are shared genes of AD and UC. They drive macrophages and microglia heterogeneous polarization, which may be potential targets for treating neural dysfunction induced by systemic inflammation and vice versa.

## 1. Introduction

Nowadays, more and more evidence shows the importance of communication between the gut and the central nervous system through the brain–gut axis. For example, inflammatory bowel disease (IBD) is a peripheral inflammatory disease that can lead to mental disorders and cognitive disorders, showing symptoms similar to Alzheimer’s disease (AD) and Parkinson’s disease (PD) [1,2,3,4,5] and even affect the hippocampal neurogenesis of the brain [6]. In a longitudinal study, it was found that there was a significant correlation between IBD and the subsequent development of dementia [7], which was consistent with the results of another Danish national cohort study from 1977 to 2018, which revealed the potential role of the gut in the development of AD [8]. On the other hand, in patients with IBD, dementia is usually diagnosed at an early stage, and the risk of the disease seems to increase with the chronicity of IBD [7]. For example, a national population-based cohort study showed that the risk of neurodegenerative diseases in IBD patients was higher than that in non-IBD patients [9]. Therefore, it is necessary to study the relationship between IBD and dementia.

AD usually impairs cognition, memory, and language. The causes of AD including pathologically amyloid-β (Aβ) accumulation, neurofibrillary tangle formation, extensive neuroinflammation, synaptic toxicity, neurodegeneration, and brain dysfunction [10,11]. It is a progressive neurodegenerative disorder, with increased incidence worldwide, imposing significant economic and social burdens [12,13]. Recently, it has been discovered that AD pathogenesis and progression were influenced by immune system processes because of the genetic overlap between AD and immune-mediated diseases [14,15]. IBD, including Crohn’s disease (CD) and ulcerative colitis (UC), is characterized by abnormal immune control and the imbalance of gut microbiota [8,16,17]. Evidence shows that AD and IBD may have common pathological changes and interrelated. Our previous studies also demonstrated that in the cerebral cortex and hippocampus of dextran sulfate sodium salt (DSS)-induced colitis model mice, the activities of astrocyte and microglia have changed significantly, and the secretion of cytokines and hormones of the HPA axis abnormally changed, indicating that peripheral inflammation is significantly related to the abnormalities of the central nervous system [18,19]. However, the exact association mechanism between AD and IBD is still unclear.

In recent years, advances in sequencing technology and bioinformatics have enabled us to explore the pathogenesis of disease–disease interaction at the genetic level [20]. In our study, the published gene expression data from the GEO was used to identify the common differential expression genes (co-DEGs) in AD and UC. Bioinformatics Analysis (GSEA, GO analysis, WikiPathways analysis, PPI network analysis, and cytoHubba.) revealed peroxisome proliferator-activated receptor gamma (*PPARG*) and nitric oxide synthase 2 (*NOS2*) presented a high relationship between AD and UC. Then, the result was verified in APP/PS1 mice for AD and DSS-induced mice for UC, which improved the credibility of the hypothesis. The shared gene signatures identified here between AD and UC are expected to provide new insights into the biological mechanisms of disease association.

## 2. Results

### 2.1. PPARG, NOS2, SELE, CXCL1, and HSP90AB1 Are Hub Genes of Both AD and UC

#### 2.1.1. GSEA Analysis in AD and UC

**Figure 1:**
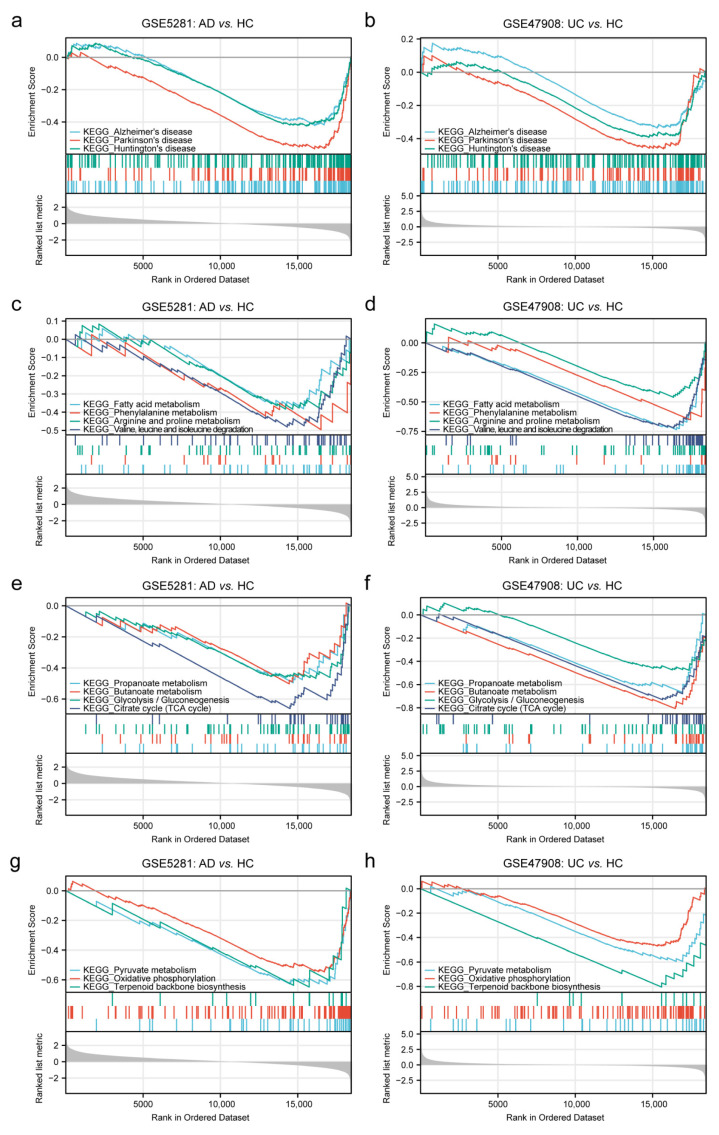
GSEA for the sample with GSE5281 (AD) and GSE47908 (UC). (**a**,**b**) GSEA analysis revealed that the genes of GSE5281 (AD) and GSE47908 (UC) were both enriched in terms of human diseases. (**c**–**h**) GSEA analysis revealed that the genes of GSE5281 (AD) and GSE47908 (UC) were both enriched in terms of metabolism.

The GSEA KEGG pathway analysis showed that 50 pathways achieved statistically significant enrichment in AD, and 71 pathways achieved statistically significant enrichment in UC (Figure S1a,b). We further screened for the KEGG pathways with consistent trends in AD and UC, and 21 common pathways were found (Table S1). In the category of KEGG, metabolism accounted for the most significant proportion (52.38%, 11/21), during Human Diseases and Organismal Systems accounted for 14.29% (Figure S1c). Interestingly, it was found that in “Human Diseases”, “Alzheimer’s disease”, “Parkinson’s disease”, and “Huntington’s disease” were significantly enriched, which belong to neurodegenerative diseases (Figure 1a,b). The categories of KEGG Metabolism include “Fatty acid metabolism”, “Phenylalanine metabolism”, “Arginine and proline metabolism”, “Valine, leucine and isoleucine degradation”, “Propanoate metabolism”, “Butanoate metabolism”, “Glycolysis/Gluconeogenesis”, “Citrate cycle (TCA cycle)”, “Pyruvate metabolism”, “Oxidative phosphorylation”, and “Terpenoid backbone biosynthesis” (Figure 1c–h).

#### 2.1.2. Identified Co-DEGs in AD and UC

**Figure 2:**
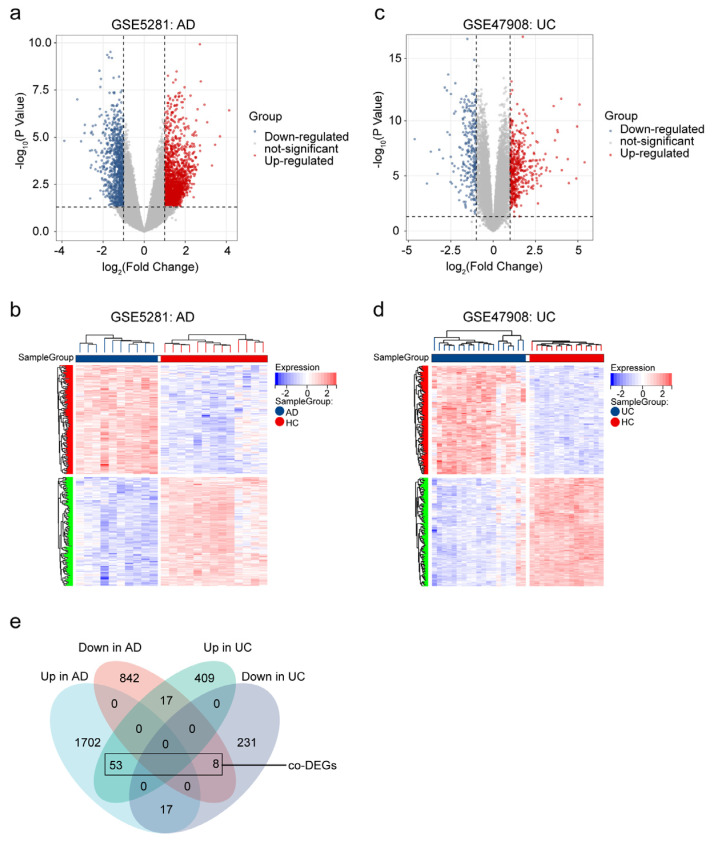
Identified co-DEGs in AD and UC. (**a**) Volcano plots of DEGs from GSE5281 (AD). (**b**) Heatmap of DEGs from GSE5281 (AD). (**c**) Volcano plots of DEGs from GSE47908 (UC). (**d**) Heatmap of DEGs from GSE47908 (UC). (**e**) Venn diagram of co-DEGs extracted from DEGs of AD and DEGs of UC.

Based on GSE5281, 2639 DEGs (1772 upregulated DEGs and 867 downregulated DEGs) were identified between AD patients and healthy controls (Figure 2a,b). Moreover, there were 735 DEGs identified between UC patients and healthy controls based on GSE47908 (479 upregulated DEGs, and 256 downregulated DEGs) (Figure 2c,d). Finally, we identified 61 co-DEGs with consistent trends in AD and UC (*p* < 0.001 using Fisher’s exact test) (Figure 2e).

#### 2.1.3. Enrichment Analysis in Co-DEGs

**Figure 3:**
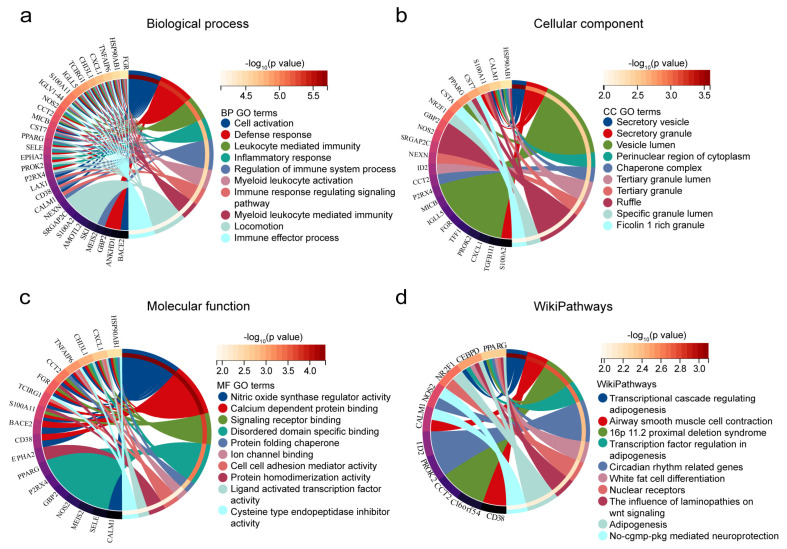
Functional annotation for co-DEGs. (**a**) Biological process, (**b**) cellular component, (**c**) molecular function, and (**d**) WikiPathways of the co-DEGs.

To further investigate the biofunctions of co-DEGs, GO and WikiPathways enrichment analysis was performed. For this set, the top 10 enriched BP GO terms were “Cell activation”, “Defense response”, “Leukocyte mediated immunity”, “Inflammatory response”, “Regulation of immune system process”, “Myeloid leukocyte activation”, “Immune response regulating signaling pathway”, “Myeloid leukocyte mediated immunity”, “Locomotion”, and “Immune effector process” (Figure 3a). With regard to CC GO terms, “Secretory vesicle”, “Secretory granule”, “Vesicle lumen”, “Perinuclear region of cytoplasm”, “Chaperone complex”, “Tertiary granule lumen”, “Tertiary granule”, “Ruffle”, “Specific granule lumen”, and “Ficolin-1-rich granule” were among the most highly enriched subcategories (Figure 3b). The top 10 GO terms in the MF category were “Nitric oxide synthase regulator activity”, “binding and transporter activity”, “Signaling receptor binding”, “Disordered domain specific binding”, “Protein folding chaperone”, “Ion channel binding”, “Cell-cell adhesion mediator activity”, “Protein homodimerization activity”, “Ligand-activated transcription factor activity”, and “Cysteine-type endopeptidase inhibitor activity” (Figure 3c). WikiPathways analysis of the 10 top ranked pathways included “Transcriptional cascade regulating adipogenesis”, “Airway smooth muscle cell contraction”, “16p 11.2 proximal deletion syndrome”, “Transcription factor regulation in adipogenesis”, “Circadian rhythm-related genes”, “White fat cell differentiation”, “Nuclear receptors”, “The influence of laminopathies on wnt signaling”, “Adipogenesis”, and “No-cgmp-pkg mediated neuroprotection” (Figure 3d).

#### 2.1.4. Constructed PPI Network, Determined Hub Genes, and ROC Analysis

**Figure 4:**
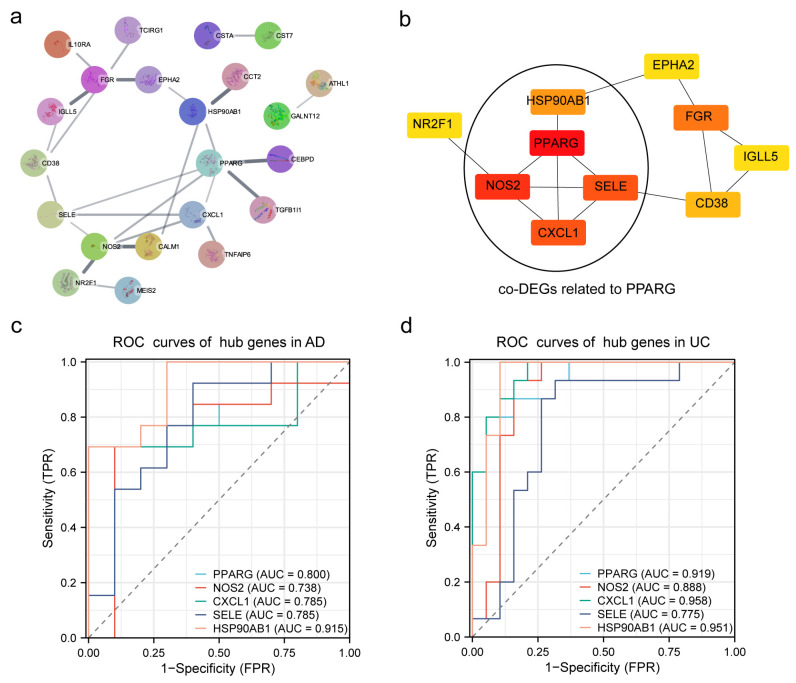
Constructed PPI network, determined hub genes, and ROC analysis and validation. (**a**) PPI network of co-DEGs. (**b**) CytoHubba to screen the top ten candidate hub genes by the MCC algorithm. ROC curves of hub genes related to *PPARG* in AD (**c**) and UC (**d**).

In order to select and further understand the hub genes between AD and UC, we first constructed a PPI network using co-DEGs in STRING (Figure 4a). Then, we used Cytoscape plug-in cytoHubba to screen the top 10 candidate hub genes by the MCC algorithm. As shown in Figure 4b, *PPARG*, *NOS2*, *SELE*, *CXCL1*, *FGR*, *HSP90AB1*, *CD38*, *EPHA2*, *NR2F1*, and *IGLL5* were picked out. Interestingly, it was found that *NOS2*, *SELE*, *CXCL1*, and *HSP90AB1* were related to *PPARG*, which was the most intensive hub gene. In Figure 4c,d, we presented representative ROC curves for the genes identified as hubs (*PPARG*, *NOS2*, *SELE*, *CXCL1*, and *HSP90AB1*). We found that the AUCs for the responsiveness of *PPARG*, *NOS2*, *SELE*, *CXCL1*, and *HSP90AB1* > 0.70 indicated moderate predictive power to AD and UC.

### 2.2. Decreasing of PPARG and HSP90AB1, and Increasing of NOS2, SELE and CXCL1 Were Found Both in AD and DSS Mice

#### 2.2.1. Induced of the Mouse Model and qRT-PCR Validation of RNA-Seq Data

**Figure 5:**
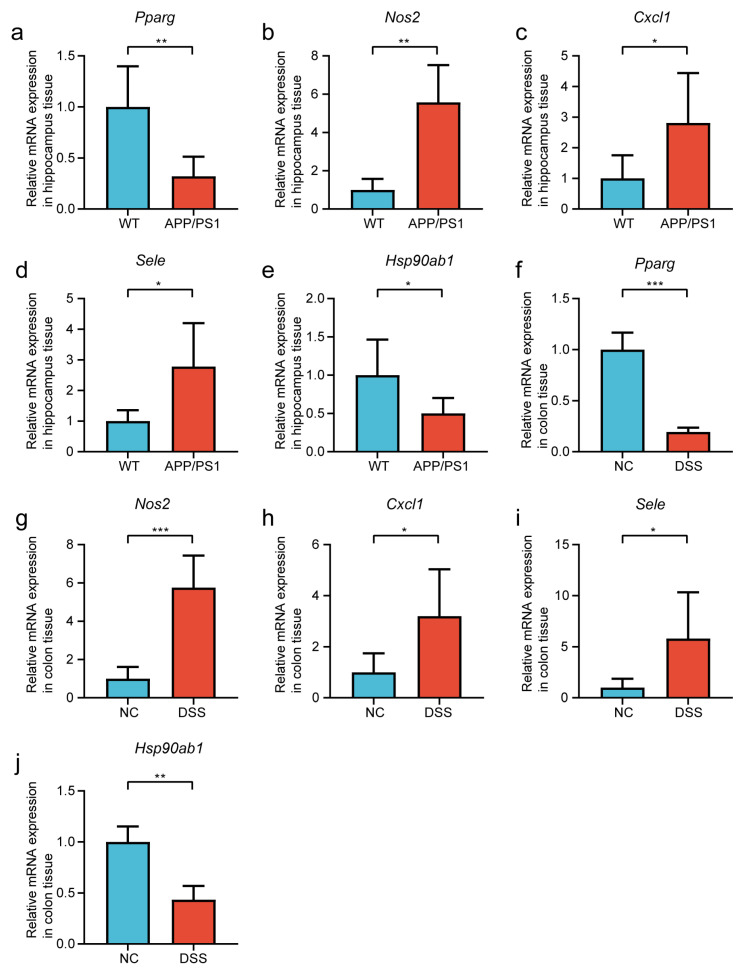
qRT-PCR results in hippocampus and colon tissues. The relative expression of *Pparg* (**a**), *Nos2* (**b**), *Cxcl1* (**c**), *Sele* (**d**), and *Hsp90ab1* (**e**) in hippocampus tissues of APP/PS1 mice and WT mice. The relative expression of *Pparg* (**f**), *Nos2* (**g**), *Cxcl1* (**h**), *Sele* (**i**), and *Hsp90ab1* (**j**) in colon tissues of DSS-induced mice and NC mice. Data are shown as mean ± SD. n = 6 in each group. * *p* < 0.05, ** *p* < 0.01, *** *p* < 0.001 (independent sample *t*-test, Mann-Whitney U test, and Welch *t*′ test).

APP/PS1 mouse model for AD and DSS-induced mice model for UC were used in the following study (Figures S2 and S3). We examined the mRNA expression levels of *PPARG*, *NOS2*, *SELE*, *CXCL1*, and *HSP90AB1* in the hippocampus of AD and colon of UC with qRT-PCR to verify the dataset’s reliability. Compared with the WT group, the expression levels of *PPARG* and *HSP90AB1* were significantly decreased, while *NOS2*, *SELE*, and *CXCL1* were significantly increased in the APP/PS1 group (Figure 5a–e). Moreover, the consistent trends were validated in the comparisons between the NC and DSS groups (Figure 5f–j).

#### 2.2.2. Protein Expression of PPAR-γ and iNOS in Both Diseases Was Confirmed by Western Blot (WB)

**Figure 6:**
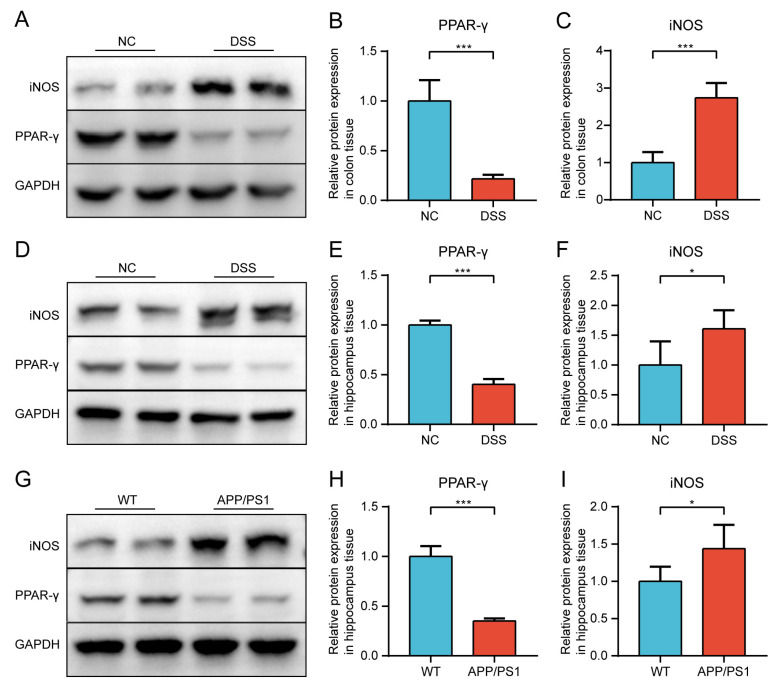
The relative expression was validated via WB. (**A**) Representative Western blot image of PPAR-γ and iNOS in colon tissues between DSS-induced mice and NC mice. (**B**) Quantification of Western blot analysis for PPAR-γ. (**C**) Quantification of Western blot analysis for iNOS. (**D**) Representative Western blot image of PPAR-γ and iNOS in hippocampus tissues between DSS-induced mice and NC mice. (**E**) Quantification of Western blot analysis for PPAR-γ. (**F**) Quantification of Western blot analysis for iNOS. (**G**) Representative Western blot image of PPAR-γ and iNOS in hippocampus tissues between APP/PS1 mice and WT mice. (**H**) Quantification of Western blot analysis for PPAR-γ. (**I**) Quantification of Western blot analysis for iNOS. Data are shown as mean ± SD. n = 6 in each group. * *p* < 0.05, *** *p* < 0.001 (independent sample *t*-test).

To further validate the hypothesis, we investigated the changes of PPAR-γ and iNOS in the colon, the hippocampus of DSS-induced mice, and the hippocampus of APP/PS1 mice by WB. Compared with the NC group, the expression levels of PPAR-γ both in colon and hippocampus tissues were significantly decreased, while iNOS in the DSS group were significantly increased (Figure 6A–F). Moreover, the consistent trends were validated in comparisons between the WT and APP/PS1 groups in hippocampus tissues (Figure 6G–I).

### 2.3. Macrophages and Microglia Tended to Be Inflammatory Polarization Both in AD and UC

**Figure 7:**
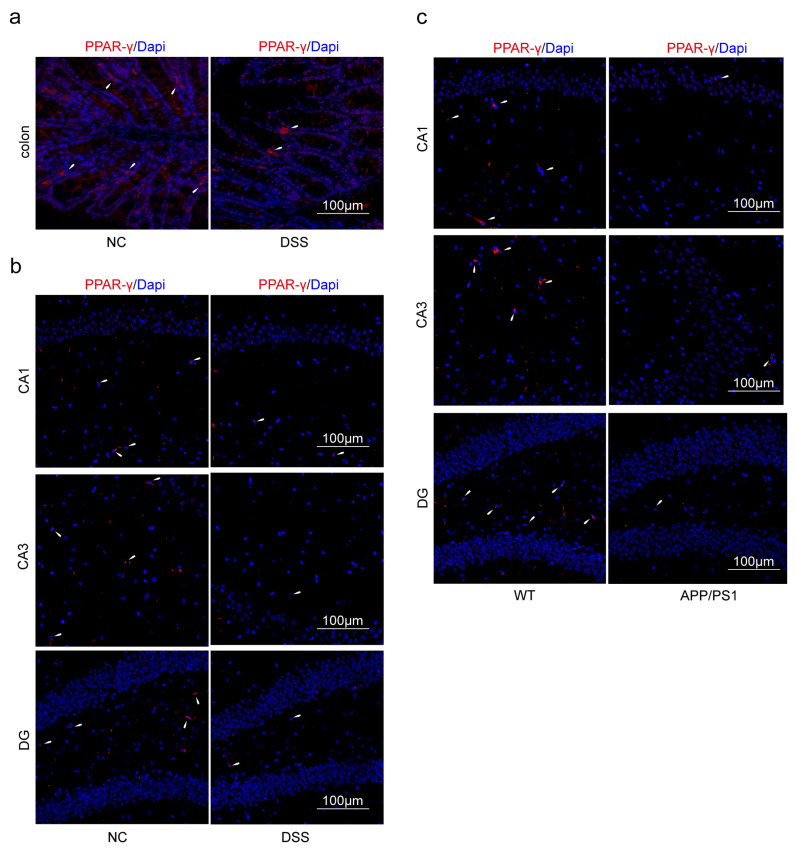
The PPAR-γ expression in different groups. (**a**) Colon was stained with anti-PPAR-γ in the NC group and DSS group. (**b**) Hippocampus was stained with anti-PPAR-γ in the NC group and DSS group. (**c**) Hippocampus was stained with anti-PPAR-γ in the WT group and APP/PS1 group. (Red expresses PPAR-γ^+^ cells, and blue expresses Dapi. The white arrow expresses PPAR-γ^+^ cells.). Magnification ×200, n = 3 in each group.

**Figure 8:**
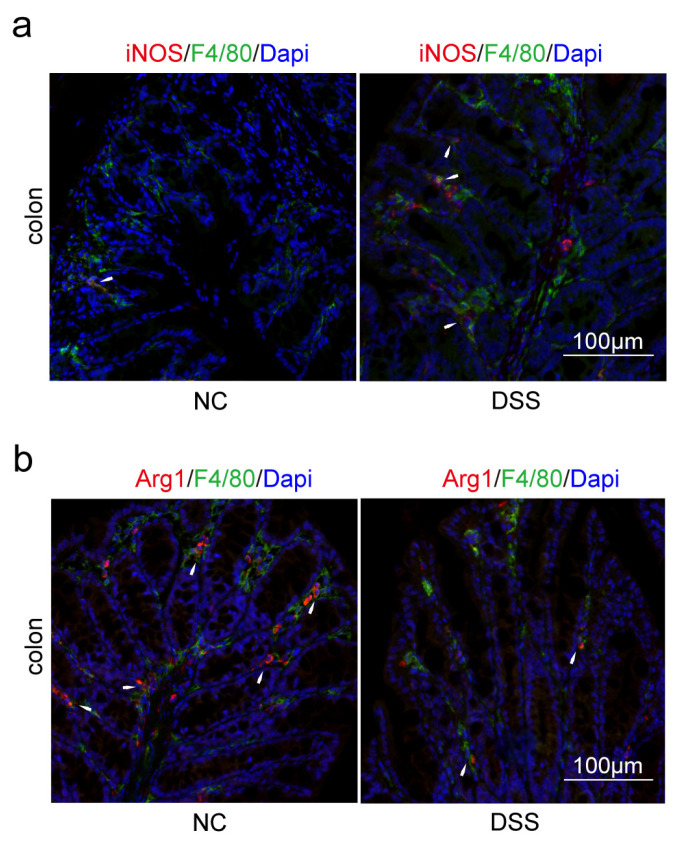
M1 macrophage polarization was promoted, and M2 macrophage polarization was suppressed in the colon of DSS-induced mice. (**a**) Macrophages of the colon were double-stained with anti-iNOS and anti-F4/80 antibodies and were observed on the fluorescent image. (Red expresses iNOS^+^ cells, green expresses F4/80^+^ cells, and blue expresses Dapi. The white arrow expresses F4/80^+^iNOS^+^ cells). (**b**) Macrophages of the colon were double-stained with anti-Arg1 and anti-F4/80 antibodies and were observed on the fluorescent image. (Red expresses Arg1^+^ cells, green expresses F4/80^+^ cells, and blue expresses Dapi. The white arrow expresses F4/80^+^Arg1^+^ cells). Magnification × 200, n = 3 in each group.

**Figure 9:**
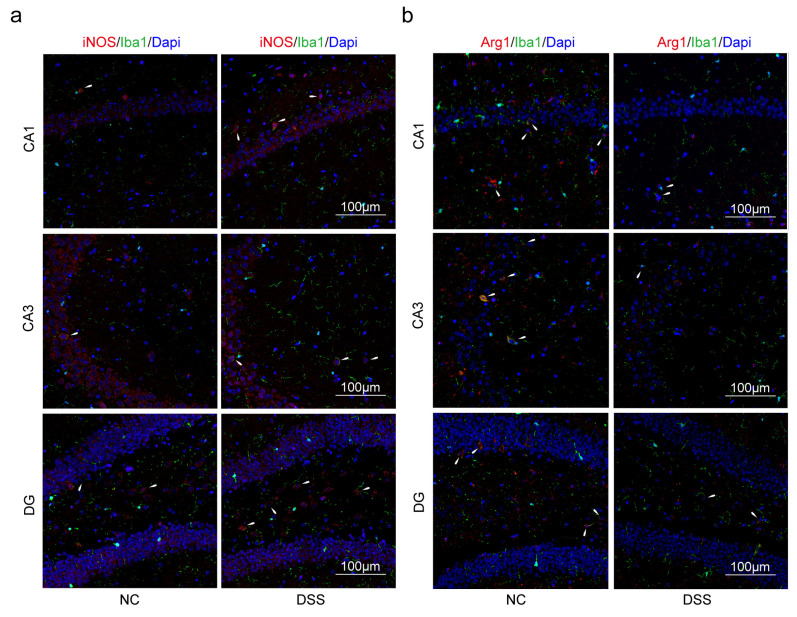
M1 microglia polarization was promoted, and M2 microglia polarization was suppressed in the hippocampus of DSS-induced mice. (**a**) Microglia of the hippocampus were double-stained with anti-iNOS, and anti-Iba1 antibodies were observed on the fluorescent image. (Red expresses iNOS^+^ cells, green expresses Iba1^+^ cells, and blue expresses Dapi. The white arrow expresses Iba1^+^iNOS^+^ cells). (**b**) Microglia of the hippocampus were double-stained with anti-Arg1, and anti-Iba1 antibodies were observed on the fluorescent image. (Red expresses Arg1^+^ cells, green expresses Iba1^+^ cells, and blue expresses Dapi. The white arrow expresses Iba1^+^Arg1^+^ cells). Magnification ×200, n = 3 in each group.

**Figure 10:**
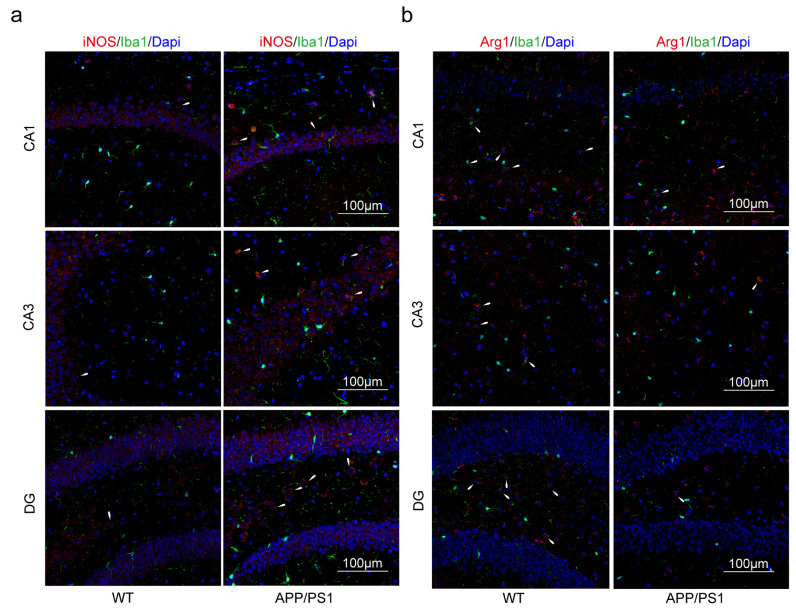
M1 microglia polarization was promoted, and M2 microglia polarization was suppressed in the hippocampus of APP/PS1 mice. (**a**) Microglia of the hippocampus were double-stained with anti-iNOS and anti-Iba1 antibody and was observed on the fluorescent image. (Red expresses iNOS^+^ cells, green expresses Iba1^+^ cells, and blue expresses Dapi. The white arrow expresses Iba1^+^iNOS^+^ cells). (**b**) Microglia of the hippocampus were double-stained with anti-Arg1 and anti-Iba1 antibodies and were observed on the fluorescent image. (Red expresses Arg1^+^ cells, green expresses Iba1^+^ cells, and blue expresses Dapi. The white arrow expresses Iba1^+^Arg1^+^ cells). Magnification ×200, n = 3 in each group.

Immunofluorescence (IF) was used to confirm that PPAR-γ in the colon and hippocampus of the DSS group was significantly reduced than that of the NC group (Figure 7a,b). Similarly, the expression of PPAR-γ in the hippocampus of the APP/PS1 group was significantly reduced than that of the WT group (Figure 7c). PPAR-γ is considered to be closely related to the M2 polarization of macrophages and microglia in some studies, while iNOS is a surface marker of M1 polarization. Therefore, we further explored the consistency of the polarization direction of colon macrophages and hippocampal microglia in two diseases. Firstly, we measured the expression of F4/80^+^iNOS^+^ and F4/80^+^Arg1^+^ cells in the colon. The double staining results revealed that the number of F4/80^+^iNOS^+^ in the DSS group was increased compared with the NC group (Figure 8a). Moreover, compared with the NC group, the expression levels of F4/80^+^Arg1^+^ cells in the colon were significantly decreased in the DSS group (Figure 8b). Furthermore, we observed that the expression of Iba1^+^iNOS^+^ cells both in the DSS group and APP/PS1 group in the hippocampus was significantly higher than those in the NC group and WT group, respectively (Figure 9a and Figure 10a). Compared with the NC group and WT group, the expression levels of Iba1^+^Arg1^+^ cells in the hippocampus were significantly decreased in the DSS group and APP/PS1 group (Figure 9b and Figure 10b).

## 3. Discussion

**Figure 11:**
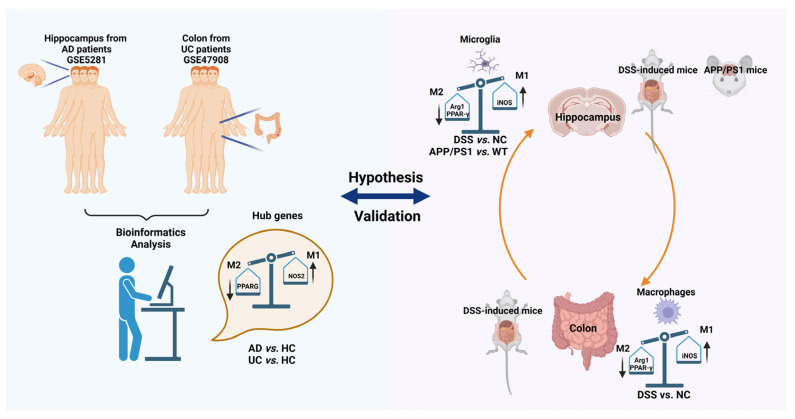
Summary and flow chart of the study. Firstly, *PPARG* and *NOS2* were the shared genes of AD and UC through bioinformatics analysis of GSE5281 (AD) and GSE47908 (UC) in the GEO database. Then, DSS-induced mice and APP/PS1 mice were used to further verify the results of bioinformatics analysis and explore the potential relationship between the two diseases. The expression level of Arg1 (a biomarker of M2-type polarization) in the macrophages of the colon and microglia of the hippocampus (DSS-induced mice) and microglia of the hippocampus (APP/PS1 mice) decreased significantly, while the expression level of iNOS (a biomarker of M1-type polarization) increased significantly were confirmed.

As shown in Figure 11, our work first confirmed that *PPARG* and *NOS2* were the shared genes of AD and UC through bioinformatics analysis of GSE5281 (AD) and GSE47908 (UC) in the GEO database. Meanwhile, we have noticed that *PPARG* and *NOS2* represent different directions in macrophages and microglia heterogeneous polarization. To further verify the results of bioinformatics analysis and explore the potential relationship between the two diseases, DSS-induced mice and APP/PS1 mice were used for our research. Interestingly, we confirmed that the expression level of Arg1 (a biomarker of M2-type polarization) in the macrophages of the colon and microglia of the hippocampus (DSS-induced mice), and microglia of the hippocampus (APP/PS1 mice) decreased significantly, while the expression level of iNOS (a biomarker of M1-type polarization) increased significantly.

In recent years, along with deep ongoing research, more and more attention has been drawn to the connection between AD and UC [7,8,9,21]. AD, the most common cause of dementia, with misfolded proteins accumulating due to the activation of microglial, oxidative stress, and the secretion of multiple cytokines, is the result of neuroinflammation from chronic systemic inflammation [22,23,24]. IBD, a disease with complex pathology that easily causes systemic inflammatory responses, was speculated that result from dysregulation of the immune response when individuals with genetic susceptibilities experience changes in their gut microbiome [7,25]. Moreover, the gut–brain axis (GBA) appears to play a critical role in neurodegenerative diseases, including AD [26]. Researchers [10,27] have shown that preventing bowel inflammation via germ-free rearing and antibiotic treatment has a positive effect on the reduction of cerebral Aβ pathology and neuroinflammation in AD model mice. A link between IBD and dementia could aid in the early detection and intervention of dementia in IBD patients and contribute to a better understanding of the long-term effects of IBD [8,28]. However, the mechanism is complex, and the correlation between the two remains unclear. For this reason, we have attempted to study the correlation between the two diseases at the genetic level to reveal the key underlying mechanism.

Global gene expression data from hippocampus tissues for AD and colon tissues for UC can help us better understand the specific pathobiology and potentially plausible biological link between the two different diseases. Firstly, the independent GSEA for GSE5281 (AD) and GSE47908 (UC) studies showed 21 common pathways between the two datasets. Interestingly, we found neurodegenerative diseases, including AD, PD, and Huntington’s disease, confirming our hypothesis that the two diseases are closely related. Meanwhile, we also found 11 pathways (52.38%, 11/21) related to metabolism, suggesting that the relationship between the two diseases may be due to changes in metabolic pathways. Next, we confirmed that 61 genes had the same tendency in the gene expression between AD and UC. The functional enrichment analysis of co-DEGs showed an inflammatory response in GO-BP and transcriptional cascade regulating adipogenesis, transcription factor regulation in adipogenesis, white fat cell differentiation, and adipogenesis in WikiPathways. To further detect the hub genes among co-DEGs, hub gene analysis was performed using the cytoHubba plug-in after constructing the PPI network. In our study, the cytohubba analysis showed that the top 10 hub genes were *PPARG*, *NOS2*, *SELE*, *CXCL1*, *FGR*, *HSP90AB1*, *CD38*, *EPHA2*, *NR2F1*, and *IGLL5* after calculating. Moreover, the most connected hub was *PPARG*, which was related to *NOS2*, *CXCL1*, *HSP90AB1*, and *SELE*. An AUC value ≥ 0.7 of the ROC suggested that the prediction performance of those hub genes was acceptable [29]. In addition, the qRT-PCR was used to validate the mRNA expression level of the five hub genes in UC and AD, confirming the reliability of the study.

Interestingly, the pair of genes *PPARG* (protein name PPAR-γ) and *NOS2* (protein name iNOS) play a crucial role in modulating the M1/M2 polarization of microglia/macrophages. PPAR-γ is an isoform of peroxisome proliferator-activated receptor (PPAR) that belongs to the nuclear receptor family of transcription factors, whose expression is abundant in white adipose tissue, brown adipose tissue, and the large intestine and spleen [30,31]. Moreover, PPAR-γ is essential for adipogenesis, energy balance, lipid biosynthesis, and adipokine production, which also validates the results of bioinformatics analysis [32]. iNOS is an inducible form of NOS, which is induced under pathological conditions [33]. As PPAR-γ with, iNOS is a central regulator of several biochemical pathways and energy metabolism, including the metabolism of glucose and lipids, based on increasing evidence [34]. We further confirmed the same trend of PPAR-γ and iNOS expression in the hippocampus of DSS-induced mice and APP/PS1 mice by WB.

Macrophages play an essential role in maintaining intestinal immune homeostasis and in the pathogenesis of IBD, and targeted therapy to them could maintain remission in IBD [35]. Macrophages are heterogeneous, plastic, and strongly influenced by the microenvironment [36]. Macrophages are classified into two major subtypes: M1 (proinflammatory or classically activated) and M2 (anti-inflammatory or alternatively activated) [37,38]. In the lamina propria of inflamed gut, M1 macrophages break down the tight junction proteins, damage the epithelial barrier, and induce epithelial cell apoptosis, leading to excessive inflammation by secreting cytokines such as IL-12, IL-23, IL-1β, TNF-α, and ROS and ultimately causing tissue damage [37,38,39,40]. Conversely, M2 macrophages promote inflammation resolution and tissue remodeling by upregulated factors such as IL-4, IL-10, CD163, CD206, and Arg1 [37,38,39,40]. According to these findings, macrophage polarization plays an increasingly important role in inflammation progression and prognosis. PPAR-γ plays a central role in promoting M2 macrophages, as demonstrated by the connection between PPAR-γ and Arg1 [40,41]. As for iNOS, it has been used to define classically activated M1 macrophages, and its associated metabolites are fundamentally involved in the intrinsic regulation of macrophage polarization and function with increasing evidence [42]. By IF, we confirmed the changes in PPAR-γ expression and macrophage polarization in colon tissues of DSS-induced mice.

In addition, via IF, we also confirmed that the trends in microglia polarization in the hippocampus of both disease mouse models were also consistent. As resident macrophages of central nervous system (CNS), microglia are thought to play a crucial role in maintaining a healthy state of the tissue environment and overcoming neuroinflammation [43,44]. The M1/M2 classification system has also emerged as a common language to discuss microglia heterogeneity across different fields, similar to macrophages [45]. It has been demonstrated that peripheral macrophages enhance infiltration of the hippocampus in DSS-induced mice, along with the increased frequency of M1-like microglia and increased release of proinflammatory cytokines [6]. Several studies have shown that in the disease process of AD, PPAR-γ plays an essential role in inhibiting the M1 polarization of microglia and promoting M2 polarization [46,47,48]. Like macrophages, iNOS is also a marker of M1 polarization in microglia and is widely recognized [49,50,51]. Moreover, lipid metabolism imbalance plays a critical role in AD pathogenesis [52]. ROS damaged lipids when pathological AD conditions, resulting in lipid peroxidation, and lipid peroxidation products were co-localized with Aβ in the brain [52]. Researchers validated 10 lipid metabolites derived from lipidomic approaches to distinguish cognitively normal individuals from those with preclinical AD in blood with an accuracy of more than 90% [53]. By causing high levels of fecal deoxycholic acid (DCA) in the colon, high fat diet (HFD) may generate macrophage activation and colonic inflammation [54]. Additionally, HFD also impaired the bioenergetics of mitochondria in the colonic epithelium, disrupted the gut epithelial barrier, and increased susceptibility to colitis [55]. Furthermore, it has been widely confirmed that lipid metabolism disorder is an important reason for the activation of macrophages/microglia [56,57,58]. In view of these, we have reason to believe that *PPARG* and *NOS2* are important biomarkers for the association of AD with UC.

In conclusion, our study proposes shared genetic signatures to illustrate the possible mechanism of AD and UC interconnection, revealing that *PPARG* and *NOS2* are shared genes of AD and UC. They drive macrophages and microglia heterogeneous polarization, which may be potential targets for treating neural dysfunction induced by systemic inflammation and vice versa.

## 4. Materials and Methods

### 4.1. GEO Dataset Selection

The hippocampus data for AD and the colonic mucosa biopsy for UC were downloaded from the GEO database (http://www.ncbi.nlm.nih.gov/geo, accessed on 5 September 2022). To better understand the mechanistic link between AD and UC, the independent datasets of GSE5281 for AD, including 10 AD tissues and 13 healthy tissues from the human hippocampus, and GSE47908 for UC, including 19 UC tissues and 15 healthy tissues from human colonic mucosa biopsies.

### 4.2. Gene Set Enrichment Analysis (GSEA)

The Kyoto encyclopedia of genes and genomes (KEGG) pathway was analyzed using the GSEA R package run in R 4.0.3 by the Molecular Signatures Database (MSigDB, v7.4, http://software.broadinstitute.org/gsea/msigdb/collections.jsp#H, accessed on 5 September 2022) [59,60].

### 4.3. Identification and Enrichment Analyses of DEGs, PPI Network Construction, and Selection of Hub Genes

DEGs were defined with a *p*-value < 0.05 and |log2 (fold change)| > 1 using the limma package [61]. To evaluate the significance of the overlap between the DEGs belonging to the AD and UC signatures, Fisher’s exact test was performed [62]. Enrichment analysis included terms from the WikiPathways database and The Gene Ontology (GO) [63,64,65]. A human PPI network was constructed based on the STRING database (medium confidence, https://string-db.org, version 11.5, accessed on 5 September 2022) [66]. Then, a cytoHubba plug-in (http://apps.cytoscape.org/apps/cytohubba, accessed on 5 September 2022) in Cytoscape (version 3.9.1) was applied to identify the top ten hub genes in the PPI network through the MCC algorithm [67,68].

### 4.4. Animals

Male 6-month-old APP/PS1 double-transgenic mice were selected as the AD mice model for verification of AD data from human samples, and the same-age C57BL/6J (wild-type, WT) mice were used as a negative control. All mice were obtained from Beijing Weishang Lituo Technology Co., Ltd. (Beijing, China, SCXK (Jing) 2016-0009). At the same time, male C57BL/6J mice (26 ± 2 g) were obtained from GemPharmatech Co., Ltd. (Chengdu, China, SCXK (Chuan) 2020-034) for verification of UC data from human samples. The mice were randomized into the normal control (NC) and DSS groups. Mice in the DSS group received 2.5% DSS (MP Biomedicals, Santa Ana, CA, USA) for seven days, while mice in the NC group received distilled water only [69]. In addition to body weight, food intake, water intake, rectal bleeding, survival, and stool consistency were monitored daily in the mice. In a pathogen-free enclosure, all animals were fed standard rodent chow and water ad libitum at ambient temperature (23 ± 1 °C).

### 4.5. Morris Water Maze (MWM) Test

It has been widely used to assess spatial learning and memory in mouse models of neurological disorders. Three parts were included in this test: the visible platform, the hidden platform, and the space exploration. A circular water tank (diameter × height, 90 cm × 50 cm) was filled with water at 22 ± 1 °C and divided equally into four quadrants (Chengdu Techman Software Co., Inc., Chengdu, China). About 1.5–2 cm below the water’s surface, the escape platform (which had a diameter of 9 cm and a height of 28 cm) was located on the constant quadrant. Mice were placed in a quiet environment before the formal test for at least 0.5 h to acclimatize to the environment in advance. They were trained once in each quadrant for 90 s, 4 times a day. If they did not find the platform within the 90 s trial limit, they were guided to the platform for 5–10 s. There were two days of visible platform testing, while four days of hidden platform testing were conducted with white water and an invisible platform. A record of each mouse’s escape latency and swimming distance was taken. On the seventh day, mice were placed in a random quadrant without the platform for a space exploration test. The ability to learn and memory was evaluated by the time it took to cross the platform, the distance swam, and the time swam in the target quadrant in 90 s.

### 4.6. Disease Activity Index (DAI)

Colitis DAI was calculated based on body weight loss, occult blood, and stool consistency for each mouse on a basis. Table S2 shows the scores for each subscale.

### 4.7. Hematoxylin and Eosin (HE) Staining and Periodic Acid Schiff (PAS) Staining

The colon tissues in the NC and DSS groups were collected and fixed with buffered 4% formalin for two days. The samples were sectioned at a thickness of 5 μm and stained with HE and PAS for histopathological examination using a light microscope (Olympus Corporation, Tokyo, Japan).

### 4.8. Quantitative Real-Time Polymerase Chain Reaction (qRT-PCR)

Total mRNA was extracted from colon and hippocampus tissues using MolPure^®^ TRIeasyTM Plus Total RNA Kit (YEASEN, Shanghai, China), and cDNA was prepared using Hifair^®^III 1st Strand cDNA Synthesis SuperMix for qPCR (YEASEN, Shanghai, China) as directed. After then, RT-qPCR analysis of the resulting cDNA was performed using Hieff UNICON Universal Blue qPCR SYBR Green Master Mix (YEASEN, Shanghai, China). Primer sequences are shown in Table S3. In this study, the relative expression levels of target genes were normalized to *β*-*actin* and calculated using the 2^−∆∆Ct^ method.

### 4.9. Western Blot (WB)

WB analysis was performed as previously described [70]. In order to obtain the protein extracts, mice’s colon and hippocampus tissues were treated with a lysis buffer containing ethylenediaminetetraacetic acid (EDTA)-free complete protease inhibitors. Then, proteins were subjected to 10% sodium dodecyl sulfate-polyacrylamide gels (SDS-PAGE). For the transfer of the bands, a polyvinylidene difluoride membrane was used. Membranes were blocked in 0.1% Tween-20 Tris-buffered saline (TBST) containing 5% nonfat dry milk for 2 h. The membranes were then incubated with the anti-PPAR-γ antibody (1:2000, Proteintech, Wuhan, China) or anti-iNOS antibody (1:500, Proteintech, Wuhan, China) overnight at 4 °C. Subsequently, membranes were incubated with a secondary antibody (1:5000, TRAN, Beijing, China) for 2 h at 37 °C. Normalization was performed by blotting the same membranes with an antibody against GAPDH (1:50,000, Proteintech, Wuhan, China).

### 4.10. Immunofluorescence (IF)

Sections were deparaffinized in xylene and rehydrated through graded alcohol series. After that, sections were washed three times in phosphate-buffered saline (PBS) for 5 min each, then incubated with 5% goat serum for 1 h at 37 °C. Sections were incubated with the rabbit anti-PPAR-γ, anti-iNOS (1:100, Proteintech, Wuhan, China), rabbit anti-Arg1 (1:200, Bioss, Beijing, China), rabbit anti-Aβ_1–42_, anti-P-Tau (1:200, Abcam, Waltham, MA, USA), mouse anti-Iba1 and anti-F4/80 (1:200, Bioss, Beijing, China) overnight at 4 °C. The sections were incubated with Cy3-conjugated Affinipure Goat Anti-Rabbit IgG (1:200, Proteintech, Wuhan, China) or Alexa Fluor 488-conjugated goat anti-rabbit IgG (1:200, Bioss, Beijing, China) at 37 °C for 2 h in a dark room after being washed 3 times with PBS. In the final step, the sections were rinsed 3 more times before being stained for 5 min with DAPI (Biyuntian, Shanghai, China). The sections were imaged using Pannoramic MIDI (3DHISTECH, Budapest, Hungary).

### 4.11. Statistical Analysis

Statistical analysis was performed using R (version 4.2.1; RStudio Inc., Boston, MA, USA), GraphPad Prism (version 8.0; GraphPad Software Inc., San Diego, CA, USA), and IBM SPSS Statistical Software (version 27.0; IBM SPSS Inc., Armonk, NY, USA). For correlation analysis, Spearman’s correlation was used to analyze the relationship between hub genes and immune cells. The receiver operating characteristic (ROC) curves and the curve (AUC) values were computed using the pROC R package. The Shapiro-Wilk normality test was used for the normality test. Variance homogeneity was evaluated with Levene’s test. Independent sample *t*-test, Mann-Whitney U test, Welch *t*′ test, and Kruskal-Wallis *t*-test were performed as needed. *p* < 0.05 indicated statistical significance. The results were presented as mean ± standard deviation (SD).

## Data Availability

The datasets presented in this study can be found in online repositories. The names of the repository/repositories and accession number(s) can be found below: Gene Expression Omnibus, accession number GSE5281 and GSE47908 with the corresponding queries.

## Supplementary Materials

The following supporting information can be downloaded at: https://www.mdpi.com/article/10.3390/ijms24065651/s1.
