# Supplementary material for: Shared Genes of PPARG and NOS2 in Alzheimer’s Disease and Ulcerative Colitis Drive Macrophages and Microglia Polarization: Evidence from Bioinformatics Analysis and Following Validation
Source: Int J Mol Sci. 2023 Mar 15;24(6):5651. doi: 10.3390/ijms24065651 (PMC10058634; doi:10.3390/ijms24065651)
Supplement: Supplementary file 1 [file ijms-24-05651-s001.zip › ijms-2238609-supplementary.pdf]

Table S1: The common KEGG pathways in GSEA. (AD: Alzheimer's disease, UC: ulcerative colitis, NES: Normalized enrichment score)

**Name**	**AD**		**UC**	
	NES	*p*-value	NES	*p*-value
Alzheimer's disease	-2.1231	0.0058	-1.2937	0.0440
Parkinson's disease	-2.7043	0.0049	-1.6968	0.0067
Huntington's disease	-2.1745	0.0064	-1.5092	0.0081
Proximal tubule bicarbonate reclamation	-1.7888	0.0030	-2.1557	0.0025
Fatty acid metabolism	-1.5379	0.0201	-2.2933	0.0026
Phenylalanine metabolism	-1.5233	0.0446	-1.6494	0.0214
Arginine and proline metabolism	-1.5800	0.0175	-1.4829	0.0131
Valine, leucine and isoleucine degradation	-1.9360	0.0035	-2.2834	0.0026
Propanoate metabolism	-1.8599	0.0033	-1.9949	0.0025
Butanoate metabolism	-1.9201	0.0033	-2.4480	0.0026
Glycolysis / Gluconeogenesis	-1.9994	0.0039	-1.6301	0.0084
Citrate cycle (TCA cycle)	-2.4680	0.0033	-2.1491	0.0026
Pyruvate metabolism	-2.5069	0.0034	-1.8547	0.0026
Oxidative phosphorylation	-2.6150	0.0049	-1.7364	0.0033
Terpenoid backbone biosynthesis	-1.9627	0.0027	-2.0150	0.0023
Oocyte meiosis	-1.3805	0.0341	-1.5399	0.0132
Peroxisome	-1.3778	0.0385	-1.7893	0.0064
Cytokine-cytokine receptor interaction	1.8388	0.0011	2.2462	0.0012
Hematopoietic cell lineage	1.5280	0.0089	1.7906	0.0014
Natural killer cell mediated cytotoxicity	1.3577	0.0343	1.4454	0.0150
Jak-STAT signaling pathway	1.6210	0.0024	1.4841	0.0027

Table S2: Sores of disease activity index (DAI) of colitis

Feature	Score	Description
Body weight loss	0	0%
	1	1–5%
	2	6–10%
	3	11–15%
	4	>15%
Feces status	0	Normal
	2	Loose stools
	4	Watery stool
Occult/Bloody stools	0	Normal
	2	Hemoccult positive
	4	Hematochezia with naked eyes

Table S3: qPCR primer name and sequence

Gene Name	Primers (5`–3`)
Pparg	F: TCGCTGATGCACTGCCTATG<br>R: GAGAGGTCCACAGAGCTGATT
Nos2	F: GTTCTCAGCCCAACAATACAAGA<br>R: GTGGACGGGTCGATGTCAC
Cxcl1	F: CTGGGATTCACCTCAAGAACATC<br>R: CAGGGTCAAGGCAAGCCTC
Sele	F: ATGCCTCGCGCTTTCTCTC<br>R: GTAGTCCCGCTGACAGTATGC
Hsp90ab1	F: GTCCGCCGTGTGTTCATCAT<br>R: GCACTTCTTGACGATGTTCTTGC
β-actin	F: GGCTGTATTCCCCTCCATCG<br>R: CCAGTTGGTAACAATGCCATGT

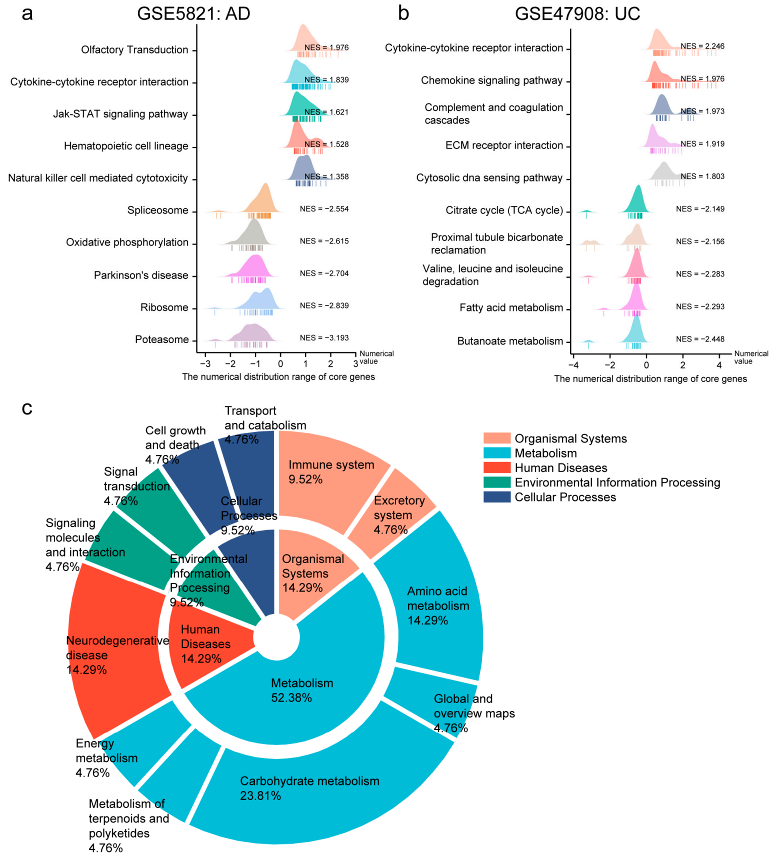

Figure S1. GSEA for the sample with GSE5281 (AD) and GSE47908 (UC). (**a**) The enriched gene sets in KEGG collection by GSE5281 (AD). (**b**) The enriched gene sets in KEGG collection by GSE47908 (UC). (**c**) The pie chart shows the proportion of different classes of KEGG pathway enriched in both GSE5281 (AD) and GSE47908 (UC). Only gene sets with $P < 0.05$ were considered significant. And only several leading gene sets were displayed in the plot. NES: Normalized enrichment score.

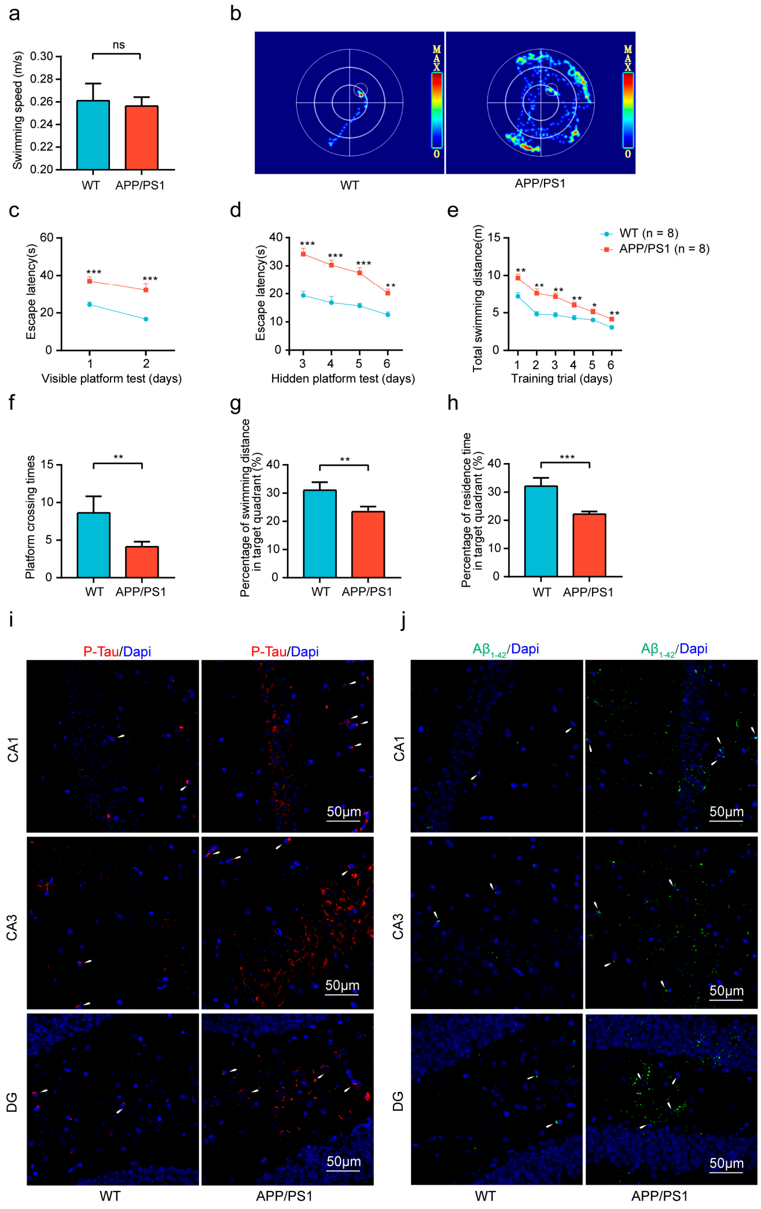

Figure S2. Establishment of the AD model mice via APP/PS1 mice. (**a**) Swimming speed. (**b**) Trajectory heat maps of the mice in the MWM test. (c) Escape latency in visible platform test. (**d**) Escape latency in hidden platform test. (**e**) Total swimming distance in training trial. (**f**) Times of crossing the platform in space exploration test. (**g**) Percentage of swimming distance in the target quadrant in space exploration test. (**h**) Percentage of residence time in the target quadrant in space exploration test. Data are shown as mean ± SD. n = 8 in each group. ns, no significance; * $P < 0.05$, ** $P < 0.01$, *** $P < 0.001$ (Independent samples t−tests and Mann−Whitney U tests). (**i**) P−tau was stained with WT and APP/PS1 groups (magnification×200, n = 3, red expresses P−tau^{+} cells and blue expresses Dapi. The white arrow expresses P−tau^{+} cells.). (**j**) $A\beta_{1-42}$ were stained with WT and APP/PS1 groups (magnification×200, n = 3, green expresses $A\beta_{1-42}^{+}$ cells and blue expresses Dapi. The white arrow expresses $A\beta_{1-42}^{+}$ cells.).

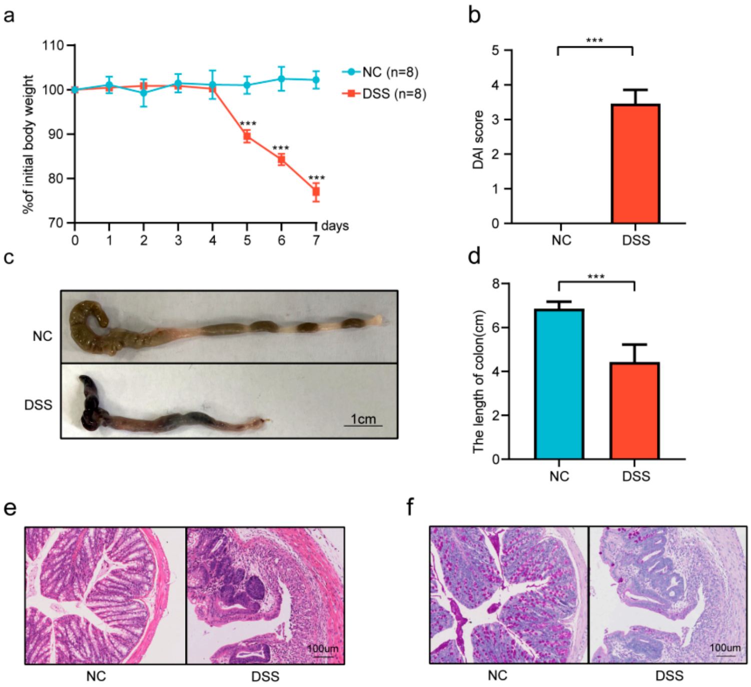

Figure S3. Establishment of the UC model mice via DSS-induced mice. (**a**) Bodyweight changes. (**b**) DAI score. (**c**) Representative images of colonic segments. (**d**) Colonic length. Data are shown as mean ± SD. n = 8 in each group. $^{***}P < 0.001$ (Independent samples t−tests). (**e**) HE staining in the colon tissues (magnification×200, n = 3). (**f**) PAS staining in the colon tissues (magnification×200, n = 3).
